# Perceptions of public primary school teachers regarding noise-induced hearing loss in South Africa

**DOI:** 10.4102/sajcd.v64i1.185

**Published:** 2017-03-28

**Authors:** Katerina Ehlert

**Affiliations:** 1 Department Speech-Language Pathology and Audiology, Sefako Makgatho Health Sciences University, South Africa

## Abstract

**Background:**

Noise-induced hearing loss (NIHL) is an increasingly growing problem in young children. This is attributed to recreational noise being the most common cause of this problem. In young children, hearing problems can delay language development and reduce academic achievements. South Africa, in particular, has limited information and protective measures regarding the conservation of hearing in school-aged children.

**Objectives:**

The main aim of the study was to determine the perception of primary school teachers regarding NIHL. The study also aimed to determine if any hearing conservation programmes are being implemented in schools and the need for training of primary school teachers regarding NIHL.

**Method:**

A survey was conducted. In order to cover the population of interest, the sampled schools in Pretoria were clustered into urban, semi-urban and rural areas.

**Results:**

The majority of the teachers included in this study are aware of NIHL and its effects. They, however, lack the necessary resources and knowledge to effectively use this information. Most (67.5%) of the teachers indicated that they have never been exposed to children with NIHL in a school setting. It was also found that the majority (84%) of the schools included in the study do not implement hearing screening and conservation programmes.

**Conclusion:**

Although the sample size was limited, the results correlate with other research in this field indicating a need for planning and implementation of hearing conservation programmes in schools, including training of teachers in order for these programmes to be effective.

## Introduction and literature review

Noise-induced hearing loss (NIHL) is a hearing impairment caused by excessive or prolonged exposure to loud sounds. The sound results in changes in the mechanism and metabolism of the inner ear as the outer hair cells (OHCs) of the cochlea are over stimulated. The endolymphatic fluids of the cochlea are also affected in the sense that the high energy transfer causes metabolic stress, which in turn results in swelling and degeneration of the balance and hearing nerve terminals that attach to the inner hair cells (IHCs). Degeneration of the hair cells of the cochlea and damage to the auditory nerve result in either temporary or permanent noise-induced threshold shifts (NITS) (Edwards, 2008).

NIHL may be sudden or progressive. With the latter, it develops gradually and is more rapid in the first decade of exposure with continuous exposure being more damaging than intermittent exposure. Sudden NIHL is caused by acoustic trauma, which results from sudden exposure to high intensity sound levels that cause instant damage to the hearing mechanism, for example, explosion or gun fire at close range (Taljaard, Leischman & Eikelboom, 2013).

Apart from the loss of hearing sensitivity, two other common symptoms related to NIHL exist. One is tinnitus or ringing in the ears and the other is temporary threshold shift (TTS).

A TTS is defined as a temporary increase in hearing thresholds after exposure to loud sounds. It can be caused by continuous noise and impulse noise. Continuous noise can be harmful when exposed to noise of 85 dB or more for more than 8 h and impulse noise of 140 dB or more. With continuous noise, for every 5 dB increase from 80 dB, exposure time halves, for example, 80 dB = 16 h, 85 dB = 8 h [South African National Standards (SANS), 2004, p. 10083]. Following exposure to a period of loud sounds, a ‘temporary’ mild hearing loss (e.g., after a long flight or bus journey, or following a loud music concert) may be present. During this period, everything sounds quieter. Immediately after exposure, the thresholds may consistently increase by as much as 40 dB. However, the thresholds return to normal after a recovery time (Harrison, 2008; Plack, 2014). The TTS usually disappears within 16 to 48 h after exposure to loud noise.

These experiences tend not to cause concern, partly, because there appears to be a full recovery. However, it is widely recognised that repeated episodes of TTS can result in permanent changes within the cochlea and hearing loss. If the OHCs in the cochlear do not recover within the hours mentioned above, then the hearing loss becomes permanent, which is known as permanent threshold shifts (PTSs) (Biassoni et al., 2014). It is suggested that the noise exposure causing TTS alters the delicate micromechanics of the cochlea, including the linkages between hair cell stereocilia, and that the recovery of such insults may not be complete. It is, therefore, suggested that any noise exposure that leads to TTS should be avoided (Harrison, 2008). The PTS results in hearing loss that is of the sensory-neural type, usually bilateral and symmetrical (Taljaard et al., 2013). Intense sound levels do not affect the outer ear but it can affect the middle ear to a lesser degree, for example, in the case of a combination of hazardous sound levels as well as severe air pressure changes (Smeltzer, Bare, Hinkle & Cheever, 2010). Table 1 describes *what is too loud, and how loud is too loud.*

**TABLE 1:** What is too loud and how loud is too loud?

Intensity (dB)	Noise exposure
150	Fireworks
120	Ambulance siren
110	Chain saw and rock concert
105	Personal listening device at maximum level
100	Woodwork centre
95	Motorcycle
90	Power mower
85	Heavy city traffic
60	Normal conversation
40	Refrigerator humming
30	Whispered voice

*Source:* Adapted from National Institute on Deafness and Other Communication Disorders. (2012). *How loud is too loud: Bookmark.* Retrieved January 31, 2017, from http://www.nidcd.nih.gov/health/hearing/pages/noise.aspx

Tinnitus can be defined as the perception of sound without external stimuli, that is, a sensation of a ringing, buzzing or whistling sound in the ear (Edwards, 2008). Although there are a number of types of tinnitus, not all are the result of cochlear damage. It is common for tinnitus to be temporary, and it is normal to sporadically hear a brief ringing in the ear, which disappears within a few seconds. However, when chronic tinnitus is experienced after exposure to loud sounds, it is not just a warning sign, but a clear indicator of cochlear injury. The ringing sound indicates that the hair cells and neurons are actually in the process of being destroyed. These cells generate a neural injury discharge because the cell membrane breakdown causes repeated depolarisation (excitation) or uncontrolled release of neurotransmitters.

Following severe acoustic trauma, tinnitus can persist and becomes permanent. It has been suggested that the initial neural injury discharge sets up (synaptic) connections in a network of auditory neurons at a more central (cortical) brain level and that these cells continue to fire spontaneously (perhaps because a local positive feedback circuit is established or because local inhibitory neuron activity is reduced). Chronic tinnitus can be as damaging to quality of life as the effects of hearing loss (Harrison, 2008).

Individuals suffering from tinnitus could additionally present with sleep disturbances, mood swings, concentration problems, personality changes and speech recognition difficulties (Edwards, 2008). Loud sounds can also lead to frustration, stress and anger, resulting in ineffective communication as the individual will need an increased effort to hear and talk. Other effects include disturbances in executive functioning such as thinking processes and execution of tasks as well as performance effects that include but are not limited to annoyance, decreased work efficiency, inability to hear alarms and danger signals, speech intelligibility miscommunication and psychological distress (Edwards, 2008). Evidently, recreational activities that induce tinnitus should be averted.

Individuals with NIHL have poor speech perception, speech processing problems as well as reduced supra-threshold functioning (Edwards, 2008). They usually understand speech in quiet environments compared with that with the presence of background or competing noise (Edwards, 2008). The communication difficulties experienced by these individuals affect their quality of life (Levey, Fligor & Kagimbi, 2012; Ringdahl, Erikson & Anderson, 1998).

NIHL is the most common form of hearing loss after hearing loss related to age (Levey et al., 2012). Hazardous sound levels in African countries are more dominant in formal sectors and seen as the most common occupational disease. Manufacturing or mining; informal occupational sectors, for example, vehicle repairing; and non-occupational sectors, for example, environmental and leisure noise, are cited as the main causes of NIHL [Bomela, 2005; World Health Organization (WHO), 2015]. Industrial workers are known to be at more risk of acquiring NIHL than any other occupation. This was attributed to employees, employers and the public’s lack of knowledge and awareness about noise and its effects resulting in ineffective hearing conservation programmes. Most research studies have focused on occupational hearing loss rather than any other form of NIHL. This is not surprising because historically, hearing loss was known as a disease that affects the elderly only.

In terms of general population studies, a report (Niskar et al., 2001) from a large-scale American national health survey indicated that 12% to 15% of school-aged children have some hearing deficits attributable to noise exposure. In Canada, there are no large surveys that specifically address NIHL. Statistics Canada data indicate that 13% of children (up to 14 years of age) have some hearing disability, but it does not separate specific causes (Statistics Canada, 2006). The American Academy of Audiology estimates that one in eight children has sustained NIHL (American Academy of Audiology, 2008). It is estimated that 16% of teenagers in the United States have sustained NIHL (Kean, 2010). Work Safe BC (Workers Compensation Board of British Columbia) (2005), in a large survey of young workers entering the workforce, reports that over 20% have some early signs of hearing loss, but this includes all causes of hearing loss, not only NIHL. A study conducted in the United States by Henderson, Hartnick and Testa (2011) indicates a significant increase in the prevalence of NIHL in youths compared to 20 years ago. This was investigated by comparing studies on noise threshold shifts amongst US youths conducted in 1994 to 1998 and 2005 to 2006 that has shown significant increases in threshold shifts especially in males.

Recent studies have shown a significant increase in the prevalence of NIHL in youth and younger children resulting from voluntary exposure to noise (Henderson et al., 2011). Young people expose themselves to potentially damaging loud sounds during leisure activities, and NIHL is diagnosed in an increasing number of adolescents (Biassoni et al., 2014). Sources of environmental hazardous sounds that young people are exposed to include, but are not limited to, noisy toys, personal entertainment or listening devices, clubs, rock concerts, fireworks and lawn mowing (Keppler, Vinck & Dhooge 2010). Taljaard et al. (2013) presented information about the market to indicate growth of the use of personal listening devices (PLDs) in children and adolescents. Their study indicated that the popular PLD, which is the Apple iPod, has sold over 50 million within the past 5-years. As the years go by, these devices are improved with features like louder output without sacrificing battery drainage. Therefore, individuals are now able to listen to louder music for longer periods of time with the maximum sound level for many portable music players (PMPs) ranging from 80 dB to 115 dB. Different types of earphones potentially increase the output by 7 dB to 9 dB. In some cases, it is possible to reach over 120 dB (Taljaard et al., 2013).

There is a need for standards and guidelines for maximum output of toys in South Africa as certain children’s toys may exceed safe listening levels, placing children at risk for NIHL (Joubert & Ellis, 2012). Furthermore, teenagers frequently play their PLDs at louder intensities than other PLD users, without perceiving the intensity and claiming that they could not enjoy their music at lower volumes. Although some PLD users may be aware of the dangers of loud and extended listening on their hearing, social factors and status result in the listeners ignoring the possible dangers. PLDs have become an ‘urban Sherpa’, people rely on their PLDs to steer their circumstances and PLD is seen as a necessity in life (Bull, 2007, Levey et al., 2012). Guidelines have been developed to decrease the risk of NIHL from PLDs, for example, individuals should not listen for more than 1.5 h at 80% of the maximum volume when using PLDs (Fligor, 2006; Levey, Levey & Fligor, 2011; Portnuff, 2016). In summary, guidelines for appropriate listening levels and duration as well as appropriate headphone use contribute to the prevention of NIHL.

The secondary effects of NIHL greatly depend on the degree or severity of the hearing loss. Individuals with mild hearing losses may experience minimal problems related to the effects of NIHL. It is, therefore, important to prevent minimal losses from progressing. NIHL is, however, preventable (WHO, 2015). It is unfortunate that even though this is the case, the prevalence of NIHL in children still continues to rise.

For a child with NIHL, the degree of deficit is expected to be mild or moderate, rather than severe or profound. However, such a loss still creates a barrier to effective communication, especially in noisy environments such as the school classroom. Furthermore, a mild-to-moderate hearing loss may not be immediately apparent to a child (similarly to adults who do not recognise that they have age-related hearing loss). Parents and clinicians should be attentive. Measures of speech discrimination often more accurately reveal a hearing problem than the simple audiogram or hearing screening tests. Hearing loss can affect language development in young children. This cascading effect results in reduced academic achievements if information is being missed at school. For adolescents, communication difficulties can lead to social isolation. There have been reports of suicide resulting from social isolation. If hearing aid use is required, the adolescent may also have problems with the cosmetic appearance of the device or the stigma attached to wearing hearing aids. This may result in the adolescent not using the aids, or they may choose to become socially isolated or only socialise with small selected groups. This ultimately will have an influence on their quality of life (Harrison, 2008). Thus, hazardous noise levels have been reported to have a negative impact on academic performance which in turn results in increased rates of absenteeism and academic failures (Needham, Crosnoe & Muller, 2004). These occur due to all the effort that is required for one to perform simple tasks such as concentrating and listening which will affect academic performance (Needham et al., 2004).

PLDs are not the only major cause of NIHL in teenagers. Significant threshold shifts were also found in adolescents who were in schools, where they practice tasks such as welding and wood processing. Noise levels in such areas were found to range from 92 dB to 100 dB, which exceeds the recommended standard noise exposure levels of 85 dB which is time-dependent, that is, longer exposure results in more significant threshold shifts. This poses a great risk of NIHL in the learners, especially as they have limited use of hearing protectors (Paunovic, 2013).

In 2001, a study by Griest, Folmer and Martin aimed to investigate the pervasiveness of NIHL in younger children. During the study, the researcher screened 5300 children’s hearing in the United States, aged 6–19. Thirteen percent of the children were found to have some degree of NIHL (Mazzola, 2013). Another research study conducted by Martin, Sobel, Griest and Howarth (2013) indicated that out of 273 third-grade students, 97% of them were at risk for acquiring NIHL, that is, they were exposed to hazardous sound levels. A study conducted in 2006 by Professor Ray Hull of Wichita State University indicated that approximately 75% of high school seniors presented with a minimal hearing loss. Youths also have a risk of acquiring NIHL in movie theatres, as the sound levels can go as high as 118 dB. From this study, Ray (2006), cited in Mazzola (2013), concluded that the probable cause of high school children’s compromised hearing was NIHL because of prolonged exposure to elevated noise levels.

Therefore, over the short term, the effects of noise overstimulation may not be noticeable, but the accumulated effects of damaging incidents will eventually lead to significant hearing loss. It is important to highlight the redundancy of hair cells in the cochlea. There are many more sensory components than required; therefore, substantial cell loss can occur before there are clinical signs of a hearing loss. However, following repeated insults, the fixed complement of hair cells ultimately runs out. This is one reason why noise-induced damage in early years may not immediately be noticeable, but may become a problem later in life (Harrison, 2008). Therefore, the issue of NIHL concerning school-aged children is a major cause of hearing loss, and hearing impairment amongst children and teenagers is increasing mostly due to voluntary exposure to loud noise (i.e., using PLDs or attending amplified music concerts) (Daniel, 2007).

The only treatment for NIHL is hearing aids, but these do not fully restore normal hearing. This should serve as a motivation to provide substantial attention to hearing loss prevention in children (Harrison, 2008). It is evident that NIHL amongst youths is prominent, and hearing conservation programmes have an important place at schools. According to Sekhar et al. (2014), NIHL in primary school children goes undetected until later stages because hearing screening programmes used in schools are ineffective in detecting high frequency hearing losses. The hearing screening programmes were found to be more effective in detecting low frequency hearing losses as school-aged children most likely present with conductive hearing losses resulting from otitis media (Sekhar et al., 2014).

It is important that teachers are aware of the significant risks, effects of NIHL and ways in which they can prevent it. One of the studies conducted by Lass (1985) stated that teachers do not have enough knowledge about NIHL. In 1988, a further study researched about the health books that teachers use at schools, and it was found that the books do not have enough information on how NIHL should be prevented; however, they had sufficient information on the anatomy and physiology of hearing (Frager, Alan & Khan, 1988 in Mazzola, 2013). Recommendations were made 30 years ago stating that hearing conservation education programmes should be implemented in all schools in the United States, but still the programmes are not being implemented effectively. This is evident in the rise of number of children acquiring NIHL.

A survey was conducted on the effectiveness of hearing conservation programmes at schools in Florida, USA, which includes promoting and implementing hearing conservation programmes, educating the learners about the effects of loud noise on the quality of life, academic performance and social life (Mazzola, 2013). Only a few schools had successful hearing conservation education programmes. In other schools, the programmes were not successful because most teachers have limited knowledge on NIHL, and they are expected to implement the programmes and be successful (Mazzola, 2013). The Centre for Disease Control stated that teachers’ lack of awareness and knowledge regarding NIHL and its effects is the reason why the hearing conservation programmes are not successful at schools.

### Problem statement

Many research studies have been conducted internationally to investigate the prevalence and the prevention of NIHL in children; however, there is little research available on NIHL in South Africa. Considering the specific risks that are often unavoidable in South Africa, for example, exposure to excessively loud music in public transport vehicles, which is commonly a mode of transport in order to get to and from school as well as exposure at social gatherings and religious services, highlights the need for research specifically in the South African context. Current international research focuses more on setting standards and guidelines for PLD use. In South Africa, research studies regarding the prevalence of NIHL in youths are extremely limited. A study conducted in 2005 in Gauteng, South Africa, on teachers’ attitudes about hearing loss indicated that teachers do not have adequate knowledge, skills and training for effective implementation of hearing conservation programmes (Pottas, 2005). No further studies focusing on NIHL in youths have been performed. The WHO (2015) states that:

Children and adolescents must be educated about the possible dangers of exposure to loud sounds from the misuse of personal listening devices and encouraged to develop safe listening habits. Such information should be part of the health education curriculum and also be taught as part of music and dance classes. (p. 15)

The lack of research in South Africa highlights the need to determine the knowledge and perceptions of primary school teachers regarding NIHL, and hearing conservation is vital in order to identify possible solutions for effective implementation of hearing conservation programmes in schools. It may also create awareness that teachers and audiologists should work together for identification and management of learners with NIHL and also in promoting NIHL prevention programmes at schools. Healthy behaviours are more easily established during childhood compared with adulthood. It would, therefore, be better for children to grow up in an educational environment whereby health promotion is emphasised in order to change listening habits and attitudes. This can only be achieved if teachers are aware of NIHL as well as its effects.

## Material and methods

### Overview

A descriptive research design was used for the purpose of this study, that is, a quantitative study whereby researchers seek to obtain insight and answer questions that the researcher has about the population of interest (De Vos, Strydom, Fouche & Delport, 2002). This research design was applicable to the research study as the researchers wanted to gain insight into teachers’ knowledge regarding NIHL, without any attempt to change this. Data were collected with the use of a survey.

The main aim of this study was to determine the perception of primary school teachers (from Grade 5–7) in Pretoria, regarding NIHL. The study also aimed to determine what and if any hearing conservation programmes are being implemented in schools and the need for training and education of primary school teachers regarding NIHL and hearing conservation.

### Participants

A combination of purposive sampling (a form of non-probability sampling) and multistage sampling (i.e., division of the study population into clusters and stratifications) methods was used. The purpose of using the two methods was to increase the feasibility and efficacy in order to accomplish certain goals of the research design. From a population of 283 public primary schools in Pretoria, a number of 164 schools were the representative sample. A 5% confidence interval and 95% confidence level was used to calculate the representative sample, which is most commonly used in quantitative research. To cover the population of interest, the sampled schools were clustered into urban (Centurion, Arcadia, Pretoria West, Pretoria Central and Pretoria East), semi-urban (Ga-Rankuwa) and rural areas (Hammanskraal) with each cluster consisting of 55 schools. The selection criteria included qualified teachers, educating students in Grade 5–7. As most of the literature regarding NIHL in youths has focused on teenagers, targeting an age group before adolescence was deemed appropriate. All teachers that complied with the inclusion criteria were given the opportunity to participate in the study.

### Materials

Semi-structured questionnaires were used. These are the kind of questionnaires that include both close- and open-ended questions. The questionnaires were adapted from two studies. The first study was that conducted by Taljaard et. al. (2013) about PLDs and the prevention of NIHL in children. The second one was that conducted by Mazzola (2013) about the minority adolescent perceptions of perceived risk of hearing loss and hearing conservation. The questions were divided into four sections for the following purposes: Section A: to obtain demographic information; Section B: to obtain information regarding the perception and knowledge of teachers regarding NIHL; Section C: to obtain information regarding the perception and knowledge of teachers regarding hearing conservation; and Section D: to determine the need for education and training of teachers regarding NIHL and hearing conservation. Refer to Appendix 1 for the questionnaire.

Reliability is the consistency with which a measuring instrument yields a certain result when the entity being measured has not changed (Leedy & Ormrod, 2005). The researchers made sure that the questions in the questionnaire are topic specific and straight forward. Double barrelled and leading questions or negative statements were not included. Validity refers to the extent to which the instrument (questionnaire) measures what it is supposed to measure (Leedy & Ormrod, 2005). The researchers adapted questionnaires from previous studies with relevant, clear and simple questions to increase validity of the measurement instrument. A pilot study was conducted to increase the validity and reliability of the measurement instrument. No changes were made, and the participants of the pilot reported that the questions were clear and easy to answer.

### Analysis

A statistician was consulted to assist with data analysis. Descriptive statistics (mean, standard deviation and variance) was used to analyse the different variables. The Statistical Packages for Social Sciences software was used to store and analyse the data.

### Ethical consideration

Ethical approval was granted by Sefako Makgatho Health Sciences University Research Ethics Committee (09 April 2015, SMUREC/H/93/2015:UG), Department of Basic Education and principals from the participating schools. Participants had to give written consent before completing the questionnaires and voluntary participation was emphasised. Researchers ensured confidentiality by having the participants complete the questionnaires anonymously. The semi-structured questionnaires were sent to the participating schools together with consent forms via emails, with some being hand delivered. Those that were hand delivered allowed clarification of questions and were collected immediately after being completed and some were collected a few days after completion. Those sent through emails were emailed back to the researchers.

## Results and discussion

Out of the 164 targeted schools, only 59 were able to take part in the study. The return rate was 5% (59/283), with 19 schools from the urban area, 20 from the semi-urban area and 20 from the rural area. De Vos et al. (2002) stated that an acceptable return rate is 25% from the sample size. This suggests that the return rate for this study is low and cannot be generalised to the population of teachers in Pretoria and other contexts. However, this study forms the basis for future research in this area and implementation of successful hearing conservation programmes in schools.

### Section A: Participant demographics

**FIGURE 1:**
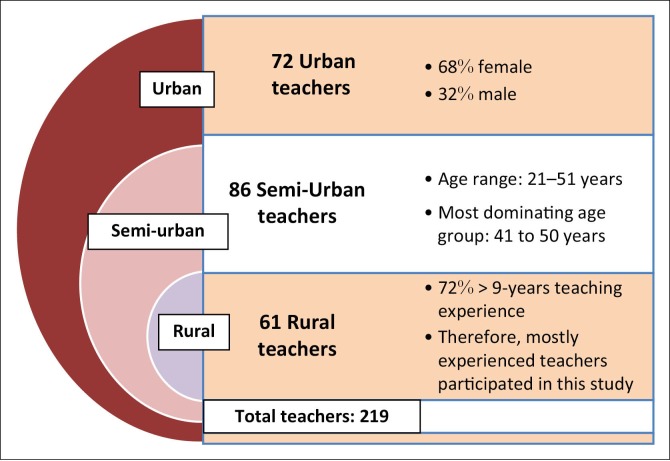
Participant demographics.

Figure 1 describes the participant demographics.

Following statistical analysis, insignificant differences were found between teachers in urban, semi-urban and rural settings. This could be due to the fact that only seven universities in South Africa provide teacher training and they are in urban settings. The results are therefore mostly reported by combining the responses from all the 219 participants.

### Section B: Perception of primary school teachers regarding noise-induced hearing loss

From the results obtained, most of the participants were able to describe hearing loss as they knew people living with hearing loss but this was not in a school setting. This is because the majority of the participants were never exposed to learners with hearing loss in a classroom. Consequently, only a minimal number (32%, *n* = 70) of the participants were able to describe the behaviours of a hearing impaired learner. According to Peel (2004), hearing impaired children are excluded from regular education schools, thus mainstreaming education is not yet global in South Africa. The results from this study, therefore, correspond with previous research as mostly teachers in special schools seem to be exposed to learners with hearing loss in the South African context. Pottas (2005) stated that although inclusive education is something that is supposed to be practised nationwide in South Africa, teachers’ negative attitudes towards hearing loss will firstly have to be addressed as they are the ones who are supposed to implement the inclusive education approach. Teachers’ negative attitudes towards the inclusion of the child with hearing loss are related to their personal experiences with hearing loss. This results in teachers excluding learners with hearing loss from regular educational settings thus resulting in fewer regular schools that allow for teaching these learners. There is a clear contrast in results of research which was conducted in Ohio, USA, which found that the majority of the respondents had classroom experience with teaching children with hearing loss. The research study was focused on teachers’ experience with hearing loss, educating children with hearing loss and their willingness to work with students with hearing impairment (Hayes, 2014).

In this study, only 26% (*n* = 57) of the participants indicated that they had experience in teaching learners with hearing loss, 74% (*n* = 162) had no experience within this area.

Furthermore, the majority (79%, *n* = 173) of the participants indicated that audiologists, teachers and parents are responsible for identifying hearing loss in learners. Intensity of sounds was indicated as the most common risk condition for NIHL, followed by the frequency of the sound and the duration of exposure to the sounds. Non-occupational noise was identified as the least common risk condition. The majority of the participants indicated that all of the following are effects of hearing loss in order of most to least importance: academic failures, cognitive difficulties, language and speech difficulties, and social problems. These results suggest that the majority of the teachers have an idea of the impact of hearing loss in children; however, their knowledge in this area is limited as social problems, language and speech problems were indicated as the least common impact of hearing loss, and it is clear that they do not understand the cascading impact of hearing loss on a child in school. According to Doyle and Dye (2002), the impact of hearing loss in children has a specific order and these effects vary with the significance of the hearing loss. This order is as follows: speech and language difficulties, academic problems and social and emotional issues. The Centre for Disease Control stated that teachers’ lack of awareness and knowledge regarding NIHL and its effects is due to lack of training. Other studies conducted by Haller and Montgomery (2004) suggest that teachers who are mostly aware of the various effects of hearing loss in children are those who have received training and thereby work in special schools.

Teachers’ knowledge regarding NIHL indicated that 3% (*n* = 6) of the participants were of the opinion that hearing loss can be reversed, 86% (*n* = 188) said NIHL can happen to anyone at any age and 10% (*n* = 22) thought that hearing loss can be reversed and can happen to anyone at any age. Only one participant (0.5%) thought that hearing loss only happens to old people. The results suggest that most of the teachers are aware that NIHL can affect any one at any age; however, a small percentage of teachers are of the opinion that NIHL can be reversed. This emphasises the need for training teachers to work with hearing impaired learners. The teachers’ awareness of the characteristics of hearing loss will improve as they gain more experience and training in working with hearing impaired children (Haller & Montgomery, 2004).

Regarding the impact of noise exposure on hearing, 98% (*n* = 216) agree that loud sounds or music can damage hearing; however, 4% (*n* = 3) of the participants were not aware of this fact. Supporting these results is a study conducted by Richburg and Goldberg (2005), who addressed teachers’ knowledge of and opinions towards minimal hearing loss. The study evaluated teachers’ perception of five myths about minimal hearing loss. Amongst the five myths, one of them was that hearing conservation programmes are not necessary for students. The results of this study indicated that the majority of teachers reported that they think hearing conservation programmes are necessary for their students as they are frequently exposed to leisure noise which can lead to hearing loss therefore suggesting that they disagree with the myth.

The impact of early identification and management of hearing loss was investigated. The majority 87% (*n* = 191) of the participants in all clusters know the importance of early identification and treatment of hearing loss. This is supported by the results of the study conducted by Hayes (2014). In this study, teachers were asked if they were willing to make accommodations and work with learners that have hearing loss. A vast majority of teachers reported ‘yes’, that is, that they were willing to work with students with hearing loss in the classroom environment and make the necessary accommodations because they know that with appropriate management and support, their learners with hearing loss would succeed just like those without hearing loss (Hayes, 2014).

**FIGURE 2:**
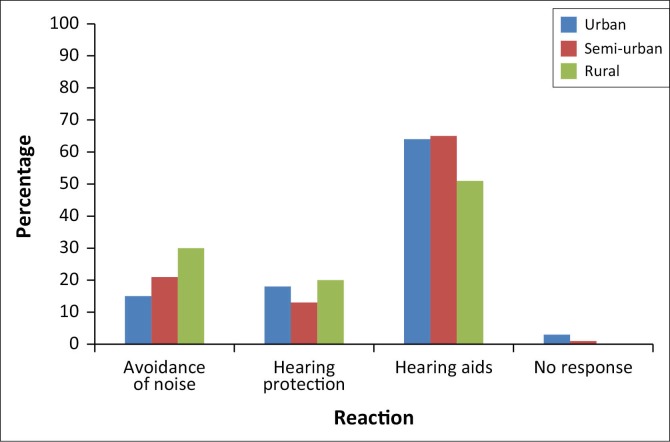
Management of noise-induced hearing loss.

Knowledge regarding the management of NIHL is represented in Figure 2.

Sixty percent of teachers have an understanding on how hearing loss is managed; however, 66% of the teachers never provide this kind of information to parents. Furthermore, it was noted that the majority of the teachers that reported that they do provide the information were mostly the ones who reported having experience in teaching learners with hearing loss in class. This coincides with the statement by Haller and Montgomery (2004) indicating that teachers who are exposed to learners with hearing loss in the school setting are most likely the ones to be involved in providing parents with specific information regarding hearing loss and associated problems.

Regarding prevention of NIHL, 69% reported that wearing hearing protection was the best way of preventing NIHL. Being fitted with hearing aids was the second most common answer. Similarly, Hayes (2014) conducted a study in Ohio that focused on knowledge of teachers regrading hearing loss and assistive listening technologies. When the teachers were asked whether they agreed that preferential seating was enough for management of hearing loss, 31% of the 45 teachers reported that they ‘disagreed’ or ‘strongly disagreed’, 36% agreed and 33% had no opinion. These results suggest that the majority of the teachers were of the opinion that preferential seating is sufficient to support children with hearing loss in the classroom setting. These results could, however, be influenced by the manner in which the questions were posed or the options that the teachers were given to answer these questions. In the same study, the teachers were asked whether they are familiar with hearing aids as a form of managing hearing loss, and the majority of the teachers (77%) reported being ‘familiar’ or ‘somewhat familiar’ with the use of amplification devices. The study conducted in 1988 in the United States regarding limited information on hearing loss in teacher health books is clearly still relevant today and applicable to the South African context, as teachers still seem to have limited knowledge regarding hearing loss, and specifically the prevention of NIHL (Frager, Alan & Khan, 1988 in Mazzola, 2013).

### Section C: Determining if any hearing conservation programmes are being implemented in schools and description thereof

From the urban areas, 19% (*n* = 14) of the participants reported that they have hearing screening programmes, 6% (*n* = 5) from the semi-urban area and 16% (*n* = 10) in the rural areas. This suggests that the urban and the rural areas have more schools with hearing screening programmes than the semi-urban areas. However, overall only 13% (*n* = 29) of the schools from all clusters do have hearing screening programmes in place. It is also not clear if these hearing screening programmes are sensitive to identifying NIHL.

According to Mazzola (2013), most schools did not have hearing conservation programmes and those ones who have programmes were not successful because most teachers have limited knowledge on NIHL from the schools that do have hearing conservation programmes running, 8% (*n* = 18) of the schools indicated that these programmes have been functional for more than 3 years. National prevention programmes have been described in the United States (Listen to Your Buds, Dangerous decibels, It is a Noisy planet), United Kingdom (Do not lose the music) and Australia (Cheers for ears, NOISE), (WHO, 2015); however, such programmes have not been initiated in South Africa.

The majority (94%, *n* = 206) of the teachers reported that they do not have the necessary equipment required to run these programmes optimally. The professionals in charge of running the hearing conservation programmes were as follows: From the urban areas, 9% (*n* = 7) of the participants indicated audiologists, 3% (*n* = 2) of them revealed speech therapists and 12% (*n* = 9) of them indicated nurses. From the semi-urban areas, 1% (*n* = 1) of the participants indicated audiologists and 2% (*n* = 2) revealed speech therapists. From the rural areas, 8% (*n* = 5) of the participants indicated audiologists and 13% (*n* = 8) revealed nurses. This suggests that the majority (19%, *n* = 25) of the school programmes are run by nurses in the urban and rural areas, with audiologists being the second most common professionals across all clusters. Under normal circumstances, hearing screening programmes should be managed by educational audiologists as they are the ones who can train other professionals to effectively take part in the programmes (Johnson & Seaton, 2012).

Educational audiologists focus on children with hearing impairment within educational settings. Duties include identification and management of hearing loss, aural (re)habilitation (hearing evaluation, language, auditory training, speech reading and speech conservation) and collaboration with teachers and school personnel in order to develop and implement individualised education plans (IEPs) for each child identified with a hearing loss. Informational and affective counselling and guidance for children, parents and teachers is a key responsibility for educational audiologists. The process of selecting, fitting and determining effectiveness of amplification is also an essential duty of educational audiologists, including participation in measurement of classroom acoustics and instructions for teachers with assistive technology. Clearly, educational audiologists have the skills necessary to collaborate with teachers in order to ensure the academic success of children with hearing loss. With the guidance and support of educational audiologists, teachers can be provided with the necessary tools and education to make the appropriate adaptations for children with hearing loss and implement successful hearing conservation programmes (Educational Audiologist Association, 2009).

### Section D: Determining if there is a need for training and education of primary school teachers regarding noise-induced hearing loss and hearing conservation

The results obtained suggest that the majority (87%, *n* = 190) of the teachers from all clusters have not received any prior training. An even higher percentage (91%, *n* = 199) of the participants indicated that they would like to be trained. These results agree with those obtained by Pottas in 2005. The study indicated that teachers do not have adequate skills and lack training for effective implementation of the hearing conversation programmes. Although teachers are educated in the methodology of providing academic education to all students, they may lack detailed information regarding the specific needs of children with hearing loss in the typical classroom. However, with the knowledge and confidence to assist children with hearing loss in the classroom, teachers can promote academic success in this population as well as support hearing conservation programmes.

The most preferred method of training and education was workshops (59%, *n* = 125); the second most common method was presentations (69%, *n* = 150) with posters (8%, *n* = 18) being the least common method. In a study conducted by Thompson, Pakulski, Price and Kleinfelder (2013), it was indicated that the most preferred method of training was the Internet (49%), followed by journals (33.2%) and workshops (30.8%) were the least preferred methods. In the study conducted by Hayes (2014), the teachers were not given options, they were only asked if they were willing to attend an in-service training and 50% of the teachers said they were unsure, 43% said yes and 7% said no. These results may be greatly influenced by the context within which the studies were done as well as the options that the teachers were given.

## Conclusions and recommendations

The main aim of this study was to determine the perceptions and knowledge of public primary school teachers regarding NIHL in Pretoria, determine if there are hearing screening programmes and also the need for education and training. The results of the study indicated that the majority of the teachers are aware of NIHL and its effects on children. However, the results also proved that most of the teachers do not have adequate information on prevention and management of NIHL. The results further indicated that most of the primary schools in Pretoria do not implement early identification programmes as well as hearing conservation programmes. School-based hearing screening protocols have typically been designed with one of the two fundamental purposes as follows: (1) an educational orientation to identify children with a hearing loss that might have negative academic consequences or (2) medically oriented programmes designed for the early identification of existing health conditions that necessitate medical referral. Therefore, a preventive approach for the purposes of identifying early signs of NIHL has not been a focus for school-based hearing screening programmes (Meinke & Dice, 2008). Although a mild high frequency sensory-neural hearing loss (SNHL) is more common in this population, this can have a severe impact on academic achievements as children can miss up to 20%–30% in a classroom environment [American Speech-Language-Hearing Association (ASHA), 2011].

The need for training and education was also highlighted as most of the teachers are willing to receive training and education regarding NIHL in the form of workshops and presentations. Aspects that should be included in hearing conservation programmes for school-aged children should include information with regard to PLDs, type of earphones or headphones used, and other potential noise dangers for children that include powered garden and domestic equipment, such as mowers and leaf blowers, as well as the recreational use of firearms. Use of hearing protectors should also be included, as well as clarification of the notion that it is not just the intensity of noise that is a problem but also the duration of exposure.

From these results, the following recommendations are made: (1) training and educating teachers about NIHL and (2) planning and implementation of hearing screening and hearing conservation programmes with an educational audiologist overseeing the programme.

### Strengths of the study

The study sample was obtained from different clusters, that is, urban areas, semi-urban areas and rural areas, representing all sectors of Gauteng. Furthermore, although the results of the study cannot be generalised, this study serves as an initial study that can be used to investigate hearing conservation programmes in other provinces and educational sectors. Additionally, the majority (85%) of the participants were able to complete the questionnaires appropriately, suggesting that the questionnaire was clear and the participants understood what the questionnaire required and can, therefore, be successfully used in future research. The anonymous aspect of this survey could be representative of the truthful responses of the teachers as there could be no consequences from their answers.

### Limitations of the study

There were insufficient participants in order to generalise the results of this study to the larger population. The limited participants could be due to method of questionnaire delivery and limited time frame of the research project. Nulty (2008) reported a trend in research of surveys from academic review from college students with lower response rates for electronic delivery of survey questionnaires when compared to paper delivery methods. Teachers may be more likely to respond to a request hand delivered to them rather than emailed to them. In future studies, private schools in all provinces of South Africa and teachers in all grades should be included in order to obtain information for a larger representation of the population. From the 258 questionnaires that were received back, only 219 of them could be used during data analysis. This is attributed to the fact that, firstly, teachers, who did not meet the inclusion criteria, completed the questionnaires and, secondly, not all the questionnaires were completed correctly. Due to the small sample size, the results of this study may not be conclusive; however, it serves as an effective pilot study that can be used for further research and to highlight the need for teacher training.

## Appendix 1

### Questionnaire

**Instructions:** Please complete the following questions to reflect your opinions as accurately as possible and to answer the questions to the best of your knowledge. Make a tick mark in check boxes for selection of options and use the space provided for answering the other questions.

Your information will be kept strictly confidential.

### Section A: Demographic information

1. Gender
  □ Male
  □ Female
2. Age
  □ 21–30
  □ 31–40
  □ 41–50
  □ 51 and above
3. How many years have you worked as a teacher?
  □ 1 month-12 months
  □ 1 year-3 years
  □ 3 years-6 years
  □ 6 years-9 years
  □ More than 9 years
4. Which grade(s) are you currently teaching?
  □ Grade 5
  □ Grade 6
  □ Grade 7

### Section B: Perception and knowledge of teachers regarding noise-induced hearing loss

1. How would you describe hearing loss? ________________________________________________
2. Has anyone you know, or have ever known had any type of communication deficits including hearing loss, cochlear implants, ringing in the ears, the use of hearing aids, etc. ________________________________________________
3. Hearing Loss
  □ Only happens to old people
  □ Never happens to kids
  □ Can be reversed
  □ Can happen to anyone at any age
4. Did you know that loud noise/music/sound can damage your hearing?
  □ Yes
  □ No
5. Who do you think is responsible for identifying noise-induced hearing loss in learners?
  □ Audiologist
  □ Doctor
  □ Speech therapist
  □ Nurses
  □ Parents
  □ Teachers
6. Which of the following conditions do you think puts a learner at a risk of having noise-induced hearing loss? (You may choose more than one answer)
  □ Loudness of sound
  □ Frequency or pitch of sound
  □ Duration of exposure
  □ Non-occupational noise e.g. firecrackers, children’s toys, sporting events, car stereo etc.
7. Which of the following do you think will be as a result of the noise-induced hearing loss? (You may choose more than one answer)
  □ Academic failures
  □ Cognitive difficulties
  □ Language and speech difficulties
  □ Social problems
8. Do you know the impact of excessive loud sounds on hearing?
  □ Yes
  □ No
9. Do you think that learners with noise-induced hearing loss that are identified and receive early treatment can develop like learners with normal hearing?
  □ Yes
  □ No
10. Did you ever have a learner or learners with hearing loss in your class?
  □ Yes
  □ No

10.1. If yes, how would you describe their behaviour? ________________________________________________
11. How do you think noise-induced hearing loss is treated?
  □ Avoidance of noise
  □ Hearing protection
  □ Hearing aids

### Section C: Perception and knowledge of teachers regarding hearing conservation

1. Does the school have any programmes aimed at identifying learners with noise-induced hearing loss?
  □ Yes
  □ No
1.1. If yes, describe the programme ________________________________________________
1.2. How long has it been implemented?
  □ 0–12 months
  □ 1–2 years
  □ More than 3 years
2. Is there a hearing screening programme in your school?
  □ Yes
  □ No
3. If yes, who is in charge of the programme? (You can choose more than one answer)
  □ Audiologist
  □ Doctor
  □ Speech therapist
  □ Nurses
  □ Parents
4. Does the school have equipment that can be used by the above mentioned people in order to identify noise-induced hearing loss?
  □ Yes
  □ No
5. Do you provide specific information to parents to help them identify possible noise-induced hearing loss?
  □ Yes
  □ No
6. Which of the following ways is a good way to prevent hearing loss?
  □ Wear hearing protection
  □ Get hearing aids
  □ Get closer to the noise
  □ Turn up the volume

### Section D: Questions to determine the need for education and training of teachers regarding noise-induced hearing loss and hearing conservation.

1. Have you ever received training and education on how to identify and work with learners who have noise-induced hearing loss?
  □ Yes
  □ No
2. Are you willing to receive more information, training and education about identifying learners with noise-induced hearing loss?
  □ Yes
  □ No
3. If yes, in what form would you like the training and education to be?
  □ Presentations
  □ Workshops
  □ Posters

## References

[CIT0001] American Academy of Audiology (2008). *Effort aims to curb number of kids who suffer from noise-induced hearing loss*. Retrieved January 31, 2017, from http://www.aufiology.org/new/pr/Pages/pr20080116.aspx

[CIT0002] American Speech-Language-Hearing Association (ASHA) (2011). *Effects of hearing loss on development*. Audiology Information Series Rockville, MD: Research Boulevard.

[CIT0003] Biassoni E.C., Serra M.R., Hinalaf M., Abraham M., Pavlik M., & Villalobo J.P. (2014). Hearing and loud music exposure in a group of adolescents at the ages of 14–15 retested at 17–18. *Noise and Health*, 16(72), 331–441. https://doi.org/10.4103/1463-1741.140515 25209043 10.4103/1463-1741.140515

[CIT0004] Bomela D. (2005). *The incidence of noise-induces hearing loss in a South African diamond mine*. Johannesburg: University of Witwatersrand.

[CIT0005] Bull M. (2007). *Sound moves: iPod culture and urban experience*. London: Routledge.

[CIT0006] Daniel E. (2007). Noise and hearing loss: A review. *Journal of School Health*, 77, 225–231. https://doi.org/10.1111/j.1746-1561.2007.00197.x 17430434 10.1111/j.1746-1561.2007.00197.x

[CIT0007] De Vos A.S., Strydom H., Fouche C.B., & Delport C.S. (2002). *Research at grass roots: For the social sciences and human service professions*. (2nd ed.). Pretoria, South Africa: Van Schaik Publishers.

[CIT0008] Doyle M., & Dye L. (2002). *Mainstreaming the student who is Deaf or Hard of hearing: A guide for professionals, teachers & parents*. San Diego, CA: Windmill Works.

[CIT0009] Educational Audiologist Association (2009). Recommended professional practices for educational audiologist. Retrieved May 3, 2016, from http://www.edaud.org

[CIT0010] Edwards A. (2008). *Characteristics of noise-induced hearing loss in gold miners*. Pretoria: University of Pretoria.

[CIT0011] Fligor B.J. (2006). ‘Portable’ music and it’s risk to hearing health. Retrieved January 31, 2017, from http://www.audiologyreview.com/issues/2006-03.asp

[CIT0012] Griest S.E., Folmer R.L., & Martin W.H. (2001). Effectiveness of dangerous decibels, a school-based hearing loss prevention program. *American Journal of Audiology*, 16(2), 165–181. https://doi.org/10.1044/1059-0889(2007/021) 10.1044/1059-0889(2007/021) 18056870

[CIT0013] Haller A.K., & Montgomery J.K. (2004). *Noise induced hearing loss in children: What educators need to know*. Circle Pines, MN: AGS Publishing.

[CIT0014] Harrison R.V. (2008). Noise-induced hearing loss in children: A ‘less than silent’ environmental danger. *Paediatric Child Health*, 13(5), 377–382. 10.1093/pch/13.5.377 PMC2532893 19412364

[CIT0015] Hayes D.N. (2014). *Survey on knowledge and attitudes of hearing loss and assistive listening technology with children*. Columbus, OH: Ohio State University, pp. 1–35.

[CIT0016] Henderson E., Hartnick C., & Testa M.D. (2011). Prevalence of noise-induce hearing-threshold shifts and hearing loss among US youths. *Paediatrics: Official Journal of The American Academy of Paediatrics*, 127, e39–e46. 10.1542/peds.2010-0926 21187306

[CIT0017] Johnson C.D., & Seaton J.B. (2012). *Educational audiology handbook*. (2nd ed.). Delmer, TX: Cengage Learning.

[CIT0018] Joubert K., & Ellis M. (2012). Noise levels of toys for children between the ages of birth and 3 years in South Africa. *South African Journal of Child Health*, 6(1), 1–9.

[CIT0019] Kean C. (2010). MP’s generation: Noise-induced hearing loss rising amongst children and adolescents. *ENT Today*. Retrieved January 31, 2017, from http://www.enttoady.org/details/article/554357/MP3_Generation_Noise-indiced_hearing_loss_rising_among_children_and_adolescents.html

[CIT0020] Keppler H., Vinck B., & Dhooge I. (2010). *Noise induced hearing loss in caused by leisure noise in youth*. New York: Nova Science Publishers, Inc.

[CIT0021] Lass N. (1985). A survey of classroom teachers and special educators knowledge of and exposure to hearing loss. *Language, Speech and Hearing Services in the Schools*, 16(3), 211–222. https://doi.org/10.1044/0161-1461.1603.211

[CIT0022] Leedy P., & Ormrod J.E. (2005). *Practical research: Planning and design*. Saddle River, NJ: Pearson Education Inc.

[CIT0023] Levey S., Fligor B.J., & Kagimbi C.G.L. (2012). The effects of noise-induced hearing loss on children and young adults. *Contemporary Issues in Communication Science and Disorders*, 39, 76–86.

[CIT0024] Levey S., Levey T., & Fligor B.J. (2011). Noise exposure estimates of urban MP3 player users. *Journal of Speech, Language and Hearing Research*, 54, 263–277. https://doi.org/10.1044/1092-4388(2010/09-0283) 10.1044/1092-4388(2010/09-0283) 20689033

[CIT0025] Martin W.H., Griest S.E., Sobel J.L., & Howarth L.C. (2013). Randomised trial of four noise-induced hearing loss and tinnitus prevention interventions for children. *International Journal of Audiology*, 52, 41–49. https://doi.org/10.3109/14992027.2012.743048 10.3109/14992027.2012.743048 23373742

[CIT0026] Mazzola H.M. (2013). *Minority adolescent perceptions of perceived risk of hearing loss and hearing conservation measures*. Honors thesis Tallahassee, FL: The Florida State University.

[CIT0027] Meinke D.K., & Dice N. (2008). Comparison of school-based hearing screening protocols and the identification of noise induced hearing loss in adolescents In *Hearing Loss: 9th International Congress on Noise as a Public Health Problem (ICBEN). ICBEN 2008., 21–25 July 2008* (pp. 141–147). Mashantucket, CT: Foxwoods.

[CIT0028] National Institute on Deafness and Other Communication Disorders (2012). *How loud is too loud: Bookmark*. Retrieved January 31, 2017, from http://www.nidcd.nih.gov/health/hearing/pages/noise.aspx

[CIT0029] Needham B.L., Crosnoe R., & Muller C. (2004). Academic failure in secondary school: The inter-related role of health problems and educational context. *Social Problems*, 51(4), 569–586. https://doi.org/10.1525/sp.2004.51.4.569 20354573 10.1525/sp.2004.51.4.569 PMC2846654

[CIT0030] Niskar A.S., Kieszak S.M., Holmes A.E., Esteban E., Rubin C., & Brody D.J. (2001). Estimated prevalence of noise induced threshold shifts among children 6 to 19 years of age: The third National Health and Nutrition Examination Survey, 1998–1994, United States. *Pediatrics*, 108, 40–43. https://doi.org/10.1542/peds.108.1.40 11433052 10.1542/peds.108.1.40

[CIT0031] Nulty D. (2008). The adequacy of response rates to online and paper surveys: What can be done? *Assessment & Evaluation in Higher Education*, 33(3), 301–314. https://doi.org/10.1080/02602930701293231

[CIT0032] Paunovic K. (2013). Noise and children’s healthy: Research in Central, Eastern and South-Eastern Europe and Newly Independent States. *Noise and Health*, 15(62), 32–41. https://doi.org/10.4103/1463-1741.107151 23412578 10.4103/1463-1741.107151

[CIT0033] Peel E.L. (2004). *Inclusive practice in South Africa: A deaf education perspective*. Johannesburg: University of Witwatersrand.

[CIT0034] Plack C.J. (2014). *The sense of hearing*. (2nd ed.). New York: Psychology Press.

[CIT0035] Portnuff C.D.F. (2016). Reducing the risk of music-induced hearing loss from overuse of portable listening devices: Understanding the problems and establishing strategies for improving awareness in adolescents. *Adolescent Health, Medicine and Therapeutics*, 7, 27–35. https://doi.org/10.2147/AHMT.S74103 10.2147/AHMT.S74103 PMC4754097 26929674

[CIT0036] Pottas L. (2005). *Inclusive education in South Africa: The teacher of the child with a hearing loss*. Pretoria: University of Pretoria.

[CIT0037] Richburg C.M., & Goldberg L.R. (2005). Teachers’ perceptions about minimal hearing loss: A role for educational audiologists. *Communication Disorders Quarterly*, 27(1), 4–19. https://doi.org/10.1177/15257401050270010301

[CIT0038] Ringdahl A., Erikson M.M., & Anderson G. (1998). Psychometric evaluation of the Gothenburg Profile for measurement of experienced hearing disability and handicap: Applications with new hearing aid candidates and experienced hearing aid users. *British Journal of Audiology*, 32(6), 375–385. https://doi.org/10.3109/03005364000000089 10064420 10.3109/03005364000000089

[CIT0039] SANS (2004). *South African National Standards:10083: The measurement and assessment of occupational noise for hearing conversation purpose*. (5th ed.). Pretoria, South Africa: Standards South Africa.

[CIT0040] Sekhar D.L., Zalewski T.R., Ghossaini S.N., King T.S., Rhoades J.A., Czarnecki B., et al. (2014). Pilot study of a high-frequency school-based hearing screen to detect adolescent hearing loss. *Journal of Medical Screening*, 21, 18–23. https://doi.org/10.1177/0969141314524565 24523012 10.1177/0969141314524565

[CIT0041] Smeltzer S.C., Bare B.G., Hinkle J.L., & Cheever K.H. (2010). *Brunner & Suddarth’s textbook of medical-surgical nursing*. Philadelphia, PA: Lippincott Williams & Wilkins.

[CIT0042] Statistics Canada (2006). *Participation and activity limitation survey: A profile of disability in Canada, 2001 – Tables*. Retrieved May 10, 2016, from http://www.statcan.ca

[CIT0043] Taljaard N.F., Leishman R.H., & Eikelboom (2013). Personal listening devices and the prevention of noise induced hearing loss in children. *Noise and Health*, 16(65), 261–268. https://doi.org/10.4103/1463-1741.113523 10.4103/1463-1741.113523 23771425

[CIT0044] Thompson A., Pakulski L., Price J., & Kleinfelder (2013). Health teacher’s perceptions and teaching practices regarding hearing loss conservation. *American Journal of Health Education*, 44(6), 335–342. https://doi.org/10.1080/19325037.2013.838917

[CIT0045] Work Safe BC, (2005) *Hearing loss prevention – Annual statistics 2005*. Retrieved May 10, 2016, from http://www.worksafebc.com

[CIT0046] World Health Organization (WHO) (2015). *Hearing loss due to recreational exposure to loud sounds: A review*. Geneva: World Health Organization.

